# MC ICP-MS *δ*^34^S_VCDT_ measurement of dissolved sulfate in environmental aqueous samples after matrix separation by means of an anion exchange membrane

**DOI:** 10.1007/s00216-015-9053-z

**Published:** 2015-10-05

**Authors:** Ondrej Hanousek, Torsten W. Berger, Thomas Prohaska

**Affiliations:** VIRIS Laboratory, Department of Chemistry, University of Natural Resources and Life Sciences Vienna, Konrad-Lorenz-Strasse 24, 3430 Tulln, Austria; Institute of Forest Ecology, Department of Forest- and Soil Sciences, University of Natural Resources and Life Sciences Vienna, Peter-Jordan-Strasse 82, 1190 Vienna, Austria

**Keywords:** ICP-MS, Multicollector, Sulfur isotope, Soil solution, Throughfall, Biogeochemistry

## Abstract

Analysis of ^34^S/^32^S of sulfate in rainwater and soil solutions can be seen as a powerful tool for the study of the sulfur cycle. Therefore, it is considered as a useful means, e.g., for amelioration and calibration of ecological or biogeochemical models. Due to several analytical limitations, mainly caused by low sulfate concentration in rainwater, complex matrix of soil solutions, limited sample volume, and high number of samples in ecosystem studies, a straightforward analytical protocol is required to provide accurate S isotopic data on a large set of diverse samples. Therefore, sulfate separation by anion exchange membrane was combined with precise isotopic measurement by multicollector inductively coupled plasma mass spectrometry (MC ICP-MS). The separation method proved to be able to remove quantitatively sulfate from matrix cations (Ca, K, Na, or Li) which is a precondition in order to avoid a matrix-induced analytical bias in the mass spectrometer. Moreover, sulfate exchange on the resin is capable of preconcentrating sulfate from low concentrated solutions (to factor 3 in our protocol). No significant sulfur isotope fractionation was observed during separation and preconcentration. MC ICP-MS operated at edge mass resolution has enabled the direct ^34^S/^32^S analysis of sulfate eluted from the membrane, with an expanded uncertainty *U* (*k* = 2) down to 0.3 ‰ (a single measurement). The protocol was optimized and validated using different sulfate solutions and different matrix compositions. The optimized method was applied in a study on solute samples retrieved in a beech (*Fagus sylvatica*) forest in the Vienna Woods. Both rainwater (precipitation and tree throughfall) and soil solution *δ*^34^S_VCDT_ ranged between 4 and 6 ‰, the ratio in soil solution being slightly lower. The lower ratio indicates that a considerable portion of the atmospherically deposited sulfate is cycled through the organic S pool before being released to the soil solution. Nearly the same trends and variations were observed in soil solution and rainwater *δ*^34^S_VCDT_ values showing that sulfate adsorption/desorption are not important processes in the studied soil.

**Electronic supplementary material:**

The online version of this article (doi:10.1007/s00216-015-9053-z) contains supplementary material, which is available to authorized users.

**Graphical abstract:**
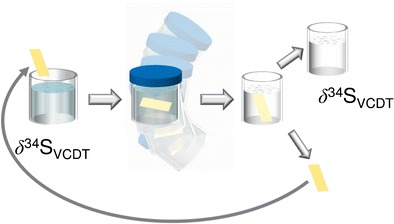
Sulfate separation by means of an anion exchange resin on a plastic membrane

## Introduction

Various processes led to the sulfur isotope (^34^S/^32^S) fractionation such as bacterial SO_4_^2−^ reduction, fractional crystallization, or evaporation of seawater [1, 2]. Regional differences in ^34^S/^32^S ratios were applied in archaeology [3], anthropology [4], or food authenticity studies [5]. Further, the isotopic system of S was applied in geochronology [6] or marine sciences [7], also with a focus on mass independent ^33^S/^32^S fractionation [8]. However, environmental studies represent the main field of application of ^34^S/^32^S analyses to shed light on the environmental sulfur cycle [1, 9, 10].

In the environment, sulfur acts as an essential nutrient for vegetation. It is a constituent of amino acids, proteins, coenzymes, or sulfolipids of plants. At the same time, sulfur (in the form of sulfate) is co-responsible for the “acid rain” phenomenon, which causes soil acidification and associated leaching of base cations from the soil [10]. Therefore, the understanding of the environmental sulfur cycle is of highest interest. Sulfur enters an ecosystem mainly in the form of sulfate (by wet and dry deposition). Sulfate is a mobile anion, which passes easily via seepage through the soil [10]. However, part of the sulfate can be taken up by plants and microbes and reduced to build organic sulfur compounds. Another part might be adsorbed on soil particles. In a reverse process, organic sulfur can be mineralized into sulfate and adsorbed sulfate can be desorbed. To a generally small extent, weathering of sulfur-bearing minerals contributes to the sulfate flow, as well [10]. Some of these processes (immobilization/mineralization, weathering) are known to result in a change of the isotopic composition of dissolved sulfate [1]. Thus, the change of the isotopic composition can serve as basis for ecological/biogeochemical modelling, helps in fertilization planning, and allows for prediction of soil recovery from acid rain effects [11]. (Throughout this publication, the term rainwater summarizes terms precipitation (rainwater above a forest canopy) and tree throughfall, i.e., precipitation after the passage through the canopy.)

The sulfur cycle can be dependent on seasonal trends and conditions, like humidity or temperature. Therefore, a long-term study of biogeochemical processes of sulfur in soil is advantageous. This requires the periodical sampling of rainwater and soil solution, considering the following analytical challenges: depending on the season, the amount of dissolved matrix elements (cations, anions, organic compounds) varies, and the amount of a water sample or the concentration of dissolved sulfate can be low (<5 mL, <0.002 mmol L^−1^, respectively). Classical method (gas source isotope ratio mass spectrometry, IRMS) requires sufficient sulfate concentration in solution, or high sample volume for precipitation of few milligrams of solid sulfate (BaSO_4_) and might therefore not be able to cope with the challenges straightforward [12].

Paris et al. have shown the capability of (multicollector) inductively coupled plasma mass spectrometry ((MC) ICP-MS) for the isotopic analysis of small amounts of dissolved sulfate [8]. When introducing only 5 to 40 nmol sulfur into the instrument, the authors reported a reproducibility (2 SD) below 0.15 ‰ for natural marine samples. Applying a matrix-matched standard, Bian et al. estimated 0.13 ‰ “external precision” (within-lab reproducibility, 2 SD) in their in-house sulfur standard [13]; Lin et al. from the same working group reached even 0.07 ‰ (2 SD) [7]. A long-term reproducibility (2 SD) of less than 0.45 ‰ was estimated for laser ablation MC ICP-MS [14, 15]. Authors using a single-collector ICP-MS reported a measurement repeatability (SD) of 0.4 ‰ in 100 ng g^−1^ S standard applying medium mass resolution [16] and 0.7 ‰ (SD) in a seawater standard in low resolution [17]. Although the latter instrumentation is still applicable for biogeochemical studies, where ^34^S/^32^S is expected to vary in the per mill range, MC ICP-MS devices are the method of choice when small isotopic differences are targeted. The main limitation during data reduction includes mainly correction for blank and instrumental isotopic fractionation (IIF). None of the authors provided combined uncertainties. However, when reporting measurement reproducibility or repeatability only, the main method limitations including correction for blank or IIF are not considered properly.

Usually, external calibration of isotopic ratios by standard-sample bracketing is applied [7, 8, 13, 14]. Correction applying internal standardization (interelemental internal IIF correction) is less common [5, 18]. The general drawback of this approach is the assumption that both elements (analyte and standard) undergo the same isotopic fractionation. In the case of sulfur, Clough has shown that the ^30^Si/^28^Si isotopic system can be used to correct measured ^34^S/^32^S ratios even in natural samples with high matrix content [18]. Other applicable correction procedures like combination of bracketing and internal standardization or double spike calibration are described, e.g., in [19].

A proper consideration of the sample composition is necessary since matrix elements can cause a significant bias in measured ^34^S/^32^S ratios. Craddock reported a shift of up to 0.7 ‰ caused by elements contained in sulfur-bearing minerals (Ca, Fe, As, Ni, Mo, Sn) [14]. Paris described the dependence of the detected sulfur signal on the Na^+^ concentration in the measured solution [8]. The effect of NaCl addition to a sulfur standard on measured ^34^S/^32^S ratio (shift by up to −2 ‰) was shown by Lin et al. [7]. To eliminate the matrix effect, Craddock and Paris used cation exchange columns (which worked well, with the exception of Mo), and Lin applied matrix-matched standards. In general, the latter is less time consuming when the matrix of studied samples can be considered as almost equal (usually within 10 % variation of the elemental content). This is the case for e.g. marine samples, where dissolved Na^+^ and Cl^−^ are the major constituents. However, the amount of dissolved matrix elements usually changes from sample to sample in rainwater and soil solution. Therefore, a separation technique is required, which is fast, robust, and reliable to allow quantitative separation and high sample throughput.

Ion exchange resins on plastic membranes have been used since the 1960s for sampling of dissolved analytes from soil [20]. When combined with a semipermeable layer, the ion exchange membrane acts as a plant root simulator (PRS). PRS is a simple and cost-saving method and, therefore, it has found a wide range of applications in soil science [21, 22]. The easiness of application, quickness, and possibility to re-use the membrane several times make the anion exchange resin on a plastic membrane an ideal candidate for sulfate separation in a high number of water samples. Kwon et al. tested an anion exchange resin placed on a polystyrene matrix for isotopic analysis of oxygen and sulfur in sulfate by IRMS [9]. They observed that the sampling method does not cause a significant isotopic fractionation of sulfur, even in the presence of other anions (competitive anion exchange). Although their method worked well, the sampled sulfate still had to be precipitated as BaSO_4_ for the subsequent isotopic analysis by IRMS. To circumvent this, a direct analysis of the sulfur isotopes by MC ICP-MS had to be validated for further application.

In this study, we demonstrate the necessity of matrix separation for reliable isotopic ratio analysis of sulfur in rainwater and soil solution. We further combined the separation by means of an anion exchange resin on plastic membrane with direct ^34^S/^32^S ratio analysis by MC ICP-MS. The tested and validated method was applied to natural rainwater and soil solution samples from a 1-year study in Austrian forest ecosystems.

## Methods

### Sample and sample preparation

All consumables were double acid washed (10 and 1 % HNO_3_*m*/*m* prepared from concentrated HNO_3_ (p.a., Merck, Darmstadt, Germany), diluted with laboratory water type I (0.055 μS cm^−1^; TKA-GenPure, Niederelbert, Germany), and rinsed with laboratory water type I. Laboratory water type I and nitric acid were further purified by using a sub-boiling distillation system (Milestone Inc., Shelton, CT, USA) and were used for dilution of standards and preparation of reagents. (NH_4_)_2_SO_4_ salts (AnalaR, VWR, Leuven, Belgium, further named as “V”; p.a., Merck, further named as “M”) were used for method development and optimization of method parameters (e.g., anion exchange time, tuning of instruments). NaHCO_3_ was used for regeneration of anion exchange membranes. Isotope certified reference materials (CRMs) IAEA-S-1, silver sulfide and IAEA-S-2, silver sulfide (both IAEA, Vienna, Austria) were used for calibration and validation of the MC ICP-MS measurement. The solid CRMs were dissolved by microwave-assisted acid digestion (Multiwave 3000, Anton-Paar, Graz, Austria): 6 mL sub-boiled HNO_3_ was added to 75 mg of a CRM. The digested material was diluted with sub-boiled water to obtain a 3.1 mmol L^−1^ S stock solution.

### Investigation of matrix effects

Dissolved LiCl, NH_4_Cl, NH_4_H_2_PO_4_, NH_4_NO_3_ (all p.a., Merck) and KCl (p.a., Sigma-Aldrich, Buchs, Switzerland) salts, single-element standards (Fe, Na (both CertiPur, Merck), Al, Ca, Mg, Mn (all Inorganic Ventures, Christiansburg, VA, USA)), and 2-propanol (Merck) were used to investigate the matrix effect of elements occurring in the investigated samples on the measured ^34^S/^32^S ratio. Investigations were performed element per element using a 60 μmol L^−1^ S solution of dissolved IAEA-S-2 certified reference material. The selection of the S concentration was based on the determined optimal S concentration for a reliable MC ICP-MS measurement (see below). Li^+^ was studied, since LiCl is often used for soil extractions. Ammonium salts were used to investigate the effect of Cl^−^ and PO_4_^3−^. Nitrate was not investigated, since 2 % HNO_3_ is the measurement matrix and thus matrix matching of standards and samples is given. 2-Propanol was used for simulation of dissolved organic compounds. The concentration of cations, anions, and organic carbon in the simulated matrix was based on the median and the maximum concentrations found in natural soil solution samples under investigation (4 and 204 μmol L^−1^ Al; 98 μmol L^−1^ and 2.5 mmol L^−1^ Ca; 2 and 159 μmol L^−1^ Fe; 56 μmol L^−1^ and 2.9 mmol L^−1^ K; 41 and 535 μmol L^−1^ Mg; 2 and 98 μmol L^−1^ Mn; 30 and 357 μmol L^−1^ Na; 550 μmol L^−1^ and 5.6 mmol L^−1^ NH_4_^+^; 50 and 705 μmol L^−1^ Cl^−^; 5 and 51 μmol L^−1^ PO_4_^3−^; 42 and 83 mmol L^−1^ C). Li concentration was based on the frequently applied extractant concentrations (1 and 10 mmol L^−1^ LiCl). In more detail, Ca, K, Li, Na, and Cl^−^ were investigated: increasing concentrations (0.1, 0.5, 0.8, 1.0, and 2.5 mmol L^−1^ Ca; 0.3, 0.6, 1.0, and 3.1 mmol L^−1^ K; 1.3, 2.5, 5.0, and 10.0 mmol L^−1^ Li; 0.2, 1.1, 2.2, 4.4, and 10.9 mmol L^−1^ Na; 0.3, 0.7, and 1.4 mmol L^−1^ Cl^−^) were added to the S reference solution and ^34^S/^32^S ratios were measured. The resulting variations of ^34^S/^32^S ratios with increasing matrix content were used to establish correlations and to estimate a lower limit of Ca/S, K/S, and Li/S ratios where no significant bias in the isotopic ratio measurements can be expected. Effects of Na^+^ and Cl^−^ were studied to relate our observations with published literature sources. The anion exchange resin membrane procedure was tested to separate the interfering elements from sulfate.

### Anion exchange on resin membranes

Commercially available anion exchange resin membranes (551642S, VWR) were cut in 2 × 3 cm pieces. Membranes were placed in 0.5 M HNO_3_ for 1 h for cleaning, rinsed with sub-boiled water, and regenerated for 4 h in a 0.5 M NaHCO_3_ (p.a., Sigma-Aldrich) solution. The regenerated membranes were rinsed with sub-boiled water and placed into 15 mL of standard solution or sample. These solutions (containing the membranes) were shaken for 16 h. The membranes were rinsed with sub-boiled water, and the adsorbed sulfate was extracted from the membrane in 15 mL 2 % HNO_3_ within 1 h of shaking.

Recovery of sulfate was tested for the SO_4_^2−^ concentration range found in our soil solution samples. The recovery was tested for actual samples, as well. Since other anions can be found in the soil solution in significant amounts, the influence of anion competition on SO_4_^2−^ exchange on the membrane was investigated: Cl^−^ and NO_3_^−^ anions in concentrations of 0.1, 1.0, 5.0, 10, and 15 mmol L^−1^ were added to the sulfate standard (0.6 mmol L^−1^ SO_4_^2-^). The kinetics of anion exchange on the membrane were investigated by placing regenerated resin membranes into a standard solution (0.9 mmol L^−1^ SO_4_^2−^) for 10 min, 30 min, and 1, 2, 4, 8, and 16 h. In order to test for sulfate preconcentration by the anion exchange membrane, we reduced the volume of the elution solution to 10 mL and to 5 mL 2 % (*m*/*m*) HNO_3_.

### Environmental samples

The study sites Jubiläumswarte, Exelberg, and Windischhütte are situated along a distance gradient (8, 10, and 13 km, respectively) from the city of Vienna, Austria, in the Vienna Woods. All sites are pure beech (*Fagus sylvatica*) stands on nutrient-rich soils with a high clay content, developed on Flysch bedrock. More details are given in [23]. Throughfall and precipitation (at an open field adjacent to each stand) samples were collected using polyethylene funnels. Soil solutions were sampled via tension lysimeters (Soilmoisture Equipment Corp., CA, USA) with a manually applied suction of −50 kPa, installed at 10, 30, and 50 cm depth in the mineral soil. Solute samples were taken monthly from May 2010 to May 2011 for ^34^S/^32^S ratio analysis. All water samples were transported to the laboratory in clean polyethylene bottles and frozen until analysis. The major quantity of the sampled soil solution is collected by the lysimeter immediately after the suction is applied. Hence, we matched rainwater chemistry of the antecedent period with chemistry of soil solution, pumped at the end of this period.

### Quantitative analyses

The content of dissolved elements in analyzed environmental samples was determined by ICP-OES (Optima 8300, PerkinElmer, Waltham, MA, USA) using external calibration. The content of dissolved anions was determined by liquid anion chromatography (ICS-900, Dionex, Sunnyvale, CA, USA). Total organic carbon was measured by TOC-L analyzer (Shimadzu, Kyoto, Japan).

Sulfate contents in standards and elemental composition of simulated matrix before and after sulfate separation were determined by single-collector ICP-MS (Element XR, Thermo Fisher Scientific, Bremen, Germany) operated at medium resolution (*R* = 4000), using external calibration and internal normalization (1 ng mL^−1^ In) prior to isotope ratio analysis.

### ^34^S/^32^S ratio analyses

A Nu Plasma HR (Nu Instruments, Wrexham, UK) MC ICP-MS was used with a desolvating nebulization system (DSN, Nu Instruments) as sample introduction system for ^34^S/^32^S ratio analyses. Measurement was performed in edge mass resolution mode (*R* ~2700), resolving spectral interferences (e.g., ^16^O^16^O, ^18^O^16^O) from the analyte signal and allowing for measurement on a flat peak shoulder at the same time. For further details on edge mass resolution and the peak shape, see, e.g., [18]. The concentration of sulfur in all samples and standards was adapted to 60 μmol L^−1^ for isotope ratio measurements. At this concentration, the best signal to noise ratio was reached. Gas flow rates and lens system voltages were optimized to reach a sensitivity of minimum 0.1 V/(μmol L^−1^) total S prior to each measurement batch. The operating parameters are summarized in Table 1. Blank correction was performed automatically by on-peak zero measurement. IIF was corrected by sample-standard bracketing. The bracketing standard IAEA-S-1 was measured before and after each sample at a concentration of 60 μmol L^−1^. All measured ratios have been expressed as delta values, relative to a VCDT ^34^S/^32^S ratio reference value according to [24]. Accuracy of measurement was assessed by measurement of IAEA-S-2 isotopic certified reference material (certified value, 22.66 ± 0.20 ‰; long-term average of measured values, 22.53 ± 0.51 ‰, 2 SD, *n* = 22) (Table 1).

**Table 1:** Operating parameters of Nu plasma HR. Gas flow rates were optimized prior to each measurement batch

RF power	1300 W
Auxiliary gas flow rate	0.91 L min^−1^
Cool gas flow rate	13 L min^−1^
DSN nebulizer pressure	~30 psi
DSN hot gas flow	~3.1 L min^−1^
DSN membrane gas flow	~0.3 L min^−1^
DSN spray chamber temperature	~112 °C
DSN membrane temperature	~118 °C
Sample uptake rate	~110 mL min^−1^
Axial mass/mass separation	33.002/0.167
Applied Faraday cup detectors	L4: ^32^S
	Ax: ^33^S
	H5: ^34^S
Measurement statistics	6 blocks
	10 measurements per block
Measurement time/sample	~10 min
Instrumental background	~1 μmol L^−1^ (total S)

### Uncertainty estimation

Quantitative measurements are expressed with an estimated uncertainty based on the standard deviation (SD) of the measurement.

The combined uncertainty of the isotope ratio measurement was calculated according to the *ISO Guide to the Expression of Uncertainty in Measurement* [25]. The uncertainties of blank correction (including correlation of blank ^34^S and ^32^S signals), ^34^S/^32^S measurement precision (SD), and IIF correction by standard-sample bracketing were propagated using the Kragten spreadsheet method [26].

## Results

### Matrix constitution and matrix effects on S isotope ratio measurements

The matrix constitution (dissolved cations, anions, and organic carbon compound concentrations) of soil solution, precipitation, and throughfall samples is summarized in Table 2.

**Table 2:** Concentration range and the median concentration of dissolved cations, anions, and organic carbon compounds in soil solution, precipitation, and throughfall samples. Number of analyzed samples, 1298

Component	Concentration/(μmol L^−1^)					
	Soil solution		Precipitation		Throughfall	
	Median	Range	Median	Range	Median	Range
Al	4	0–204	0	0–30	0	0–26
Ca	98	10–2550	23	8–315	50	15–428
Fe	2	0–159	0	0–4	0	0–7
K	56	0–2897	13	0–354	74	8–1105
Mg	41	0–535	4	4–95	21	4–140
Mn	2	0–98	0	0–18	0	0–29
Na	30	0–357	4	0–335	13	0–252
Cl^−^	51	3–705	11	3–412	14	6–370
NO_3_^−^	223	2–3242	29	3–1606	77	3–1123
PO_4_^3−^	4	2–67	4	1–27	5	1–54
SO_4_^2−^	89	2–1015	16	4–200	34	4–1289
TOC	642	100–47,000	325	183–1150	650	83–3050

**Fig. 1:**
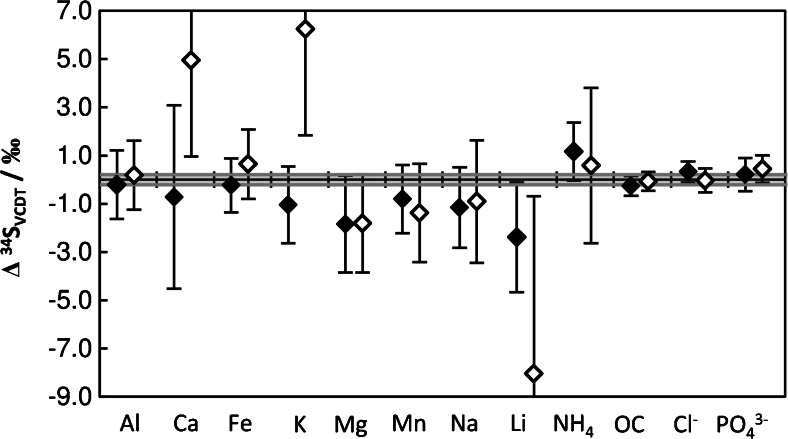
The influence of addition of matrix elements at median (*black diamonds*) and highest (*white diamonds*) concentration retrieved in soil solution samples (see Table 2) on measured *δ* ^34^S_VCDT_ values. *Δ* ^34^S_VCDT_ represents a relative shift from the reference value (*grey range*). *OC* stands for organic carbon. *Error bars* are expanded uncertainties *U* (*k* = 2). The observed increase of uncertainty is explained in following paragraphs

Since the soil solutions represent a higher matrix content among the investigated sample types, the effect of the matrix on the S isotope ratio was tested based on concentrations (the median and the maximum concentration) in these samples (see “Methods” section). Figure 1 shows the influence of cations, anions, and organic carbon on the measured sulfur isotope ratios. The values are expressed as a relative shift from the reference value (grey range).

It was observed that only Ca, K, and Li caused a significant bias of the isotopic ratio (i.e., the measured ratio differed from the reference value even under consideration of the expanded uncertainty). The influence of these elements on the analysis was investigated in more detail by adding stepwise increasing concentrations of these elements to the S standard. The resulting correlations are shown in Fig. S1 (see Electronic Supplementary Material (ESM)). The parameters of these correlations are summarized in Table 3. The repeatability of the Ca-*δ*^34^S_VCDT_ regression curve within one measurement day was chosen to test the applicability of using a mathematical model for correction of the matrix effect. The relative standard deviation of the slopes of three regression lines was 39 %.

**Table 3:** Regression curve parameters for the dependence of measured *δ* ^34^S_VCDT_ values on increasing amount of Ca, K, or Li in a S standard. Element/S rat. stands for the lowest Ca/S mass ratio already leading to a significant bias in measured *δ* ^34^S_VCDT_ ratios and K/S or Li/S mass ratio leading to imprecise (*U* > 2 ‰ (*k* = 2)) measurement

Element	Ca	K	Li
Regression type	Linear	Linear	Linear
Slope	0.146	−0.019	−0.093
*R* ^2^ factor	0.866	0.925	0.936
Repeatability	39 % (*n* = 3)	-	-
Element/S rat.	5	5	1

**Fig. 2:**
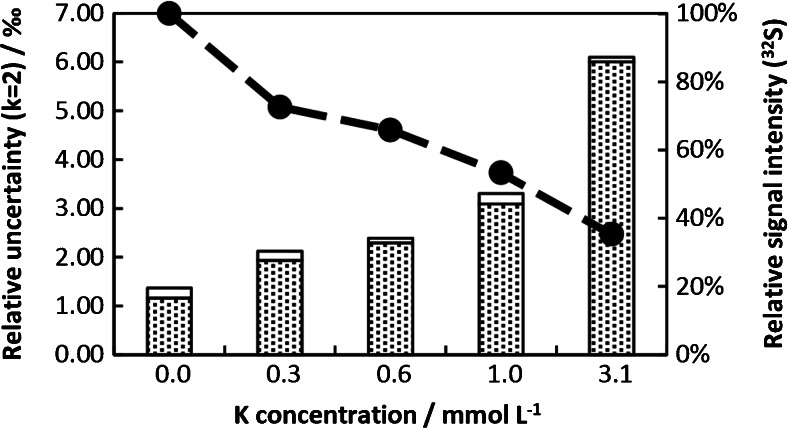
A relative decrease of the signal intensity on ^32^S (*dashed line*) and increase of the expanded uncertainty of the measurement (*bars*) with increasing concentration of K in a S standard. *White bars* show the summarized contribution of measurement precision and calibration of S isotope ratios, and *dotted bars* show the contribution of blank correction to the uncertainty

The observed decrease in the detected signal intensity of ^32^S and the increase of the combined measurement uncertainty with increasing cation concentration in the S standard is shown in Fig. 2 on the example of K. The main contributor to the uncertainty is the correction for instrumental background. Since S signal is suppressed significantly by the matrix, the contribution of the instrumental background to the total combined uncertainty increased with increasing matrix concentration. The combined measurement uncertainty increased by a factor of about 5 within the observed concentration range.

Our observations were not fully consistent with previous findings [7], where a possible bias was explained by the presence of Cl^−^ in the solution (added as NaCl) from a level of 0.3 mmol L^−1^ Cl^−^ in 0.3 mmol L^−1^ S solution. Therefore, the influence of Cl^−^ and Na^+^ on the final *δ*^34^S_VCDT_ value was investigated in more detail. No significant bias in measured *δ*^34^S_VCDT_ ratios was observed when adding up to 1.4 mmol L^−1^ Cl^−^ (added as NH_4_Cl) (Cl/S mass ratio = 25). In contrast, the addition of Na caused a significant decrease of *δ*^34^S_VCDT_ ratios of the S standard, from a level of 1.1 mmol L^−1^ (which is far above the concentration range in the investigated samples). The bias effect followed a linear (*R*^2^ = 0.974) dependence on the increasing Na concentration (see ESM Fig. S1). The addition of both Cl^−^ and Na caused an increase of the combined measurement uncertainty, since a suppression of the analyte signal was observed in both cases.

### Anion exchange on resin membranes

The efficiency of sulfate separation by the anion exchange resin on a membrane was tested for the SO_4_^2−^ concentration range of the investigated solution samples (see Table 2 and ESM Table S1). One hundred percent recovery (±1 %, SD, *n* = 14) was accomplished for all samples in a pH range of 2–11. The influence of Cl^−^ and NO_3_^−^ on the sulfate exchange efficiency was negligible at a concentration of less than 5 mmol L^−1^ (which corresponds to the concentration ranges in the investigated samples). At a Cl^−^ and NO_3_^−^ concentration of 10 and 15 mmol L^−1^, the recovery of sulfate decreased to 65 and 55 %, respectively. The kinetics were studied by using a 0.9 mmol L^−1^ sulfate standard. It was observed that sulfate from the immerse solution was exchanged quantitatively within 1 h. Addition of Ca, K, Li, or Na changed the kinetics (see ESM Table S1) leading to slower exchange rates (or lower sulfate recovery). Moreover, an enrichment factor of about 3 was obtained under routine laboratory conditions when starting with an initial volume of 15 mL and an elution volume of 5 mL (which corresponds to the volume needed for the subsequent direct isotope ratio measurement). Laboratory tests are summarized in ESM Table S1.

Quantitative matrix separation was obtained for all elements under investigation: Ca (up to 2.5 mmol L^−1^), K (up to 4.2 mmol L^−1^), Li (up to 7.2 mmol L^−1^), Na (up to 3.9 mmol L^−1^), organic carbon (up to 12.5 mmol L^−1^), and Ca (up to 2.5 mmol L^−1^).

**Fig. 3:**
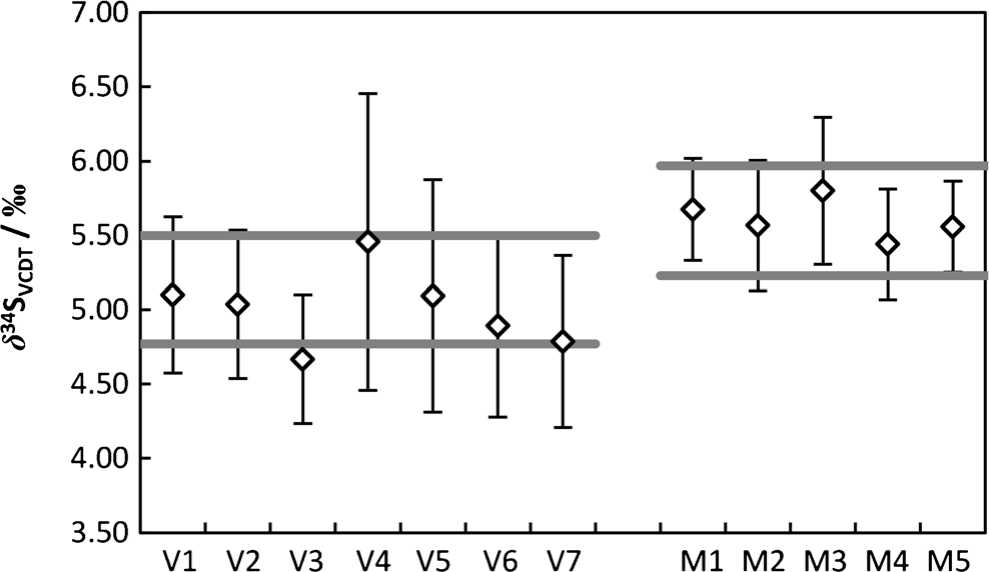
Reproducibility of the sulfate separation procedure in combination with MC ICP-MS on the example of *V1–V7* and *M1–M5* (NH_4_)_2_SO_4_ solutions. *Horizontal grey lines* show upper and lower *δ* ^34^S_VCDT_ limits of the corresponding initial solution (mean of three measurements ± *U* (*k* = 2)). *Error bars* are expanded uncertainties *U* (*k* = 2)

Isotopic ratio analysis showed no significant difference in *δ*^34^S_VCDT_ values between the initial solution and the eluate for both tested ammonium sulfate solutions (Fig. 3). Seven replicate analyses were performed for the (NH_4_)_2_SO_4_ salt “V” (V1–V7) and five for the (NH_4_)_2_SO_4_ salt “M” (M1–M5) solutions, following the procedure described in the “Methods” section. Sulfate enrichment by elution in reduced elution volume (10 or 5 mL) did not show an isotopic effect (see ESM Fig. S2). Furthermore, no effect was observed in a simulated matrix solution, when the concentration of dissolved anions (NO_3_^−^ or Cl^−^ accompanying added matrix elements) did not exceed 5 mmol L^−1^ in Ca- and K-enriched solutions (see ESM Table S1).

### Precipitation, throughfall, and soil solution samples

**Fig. 4:**
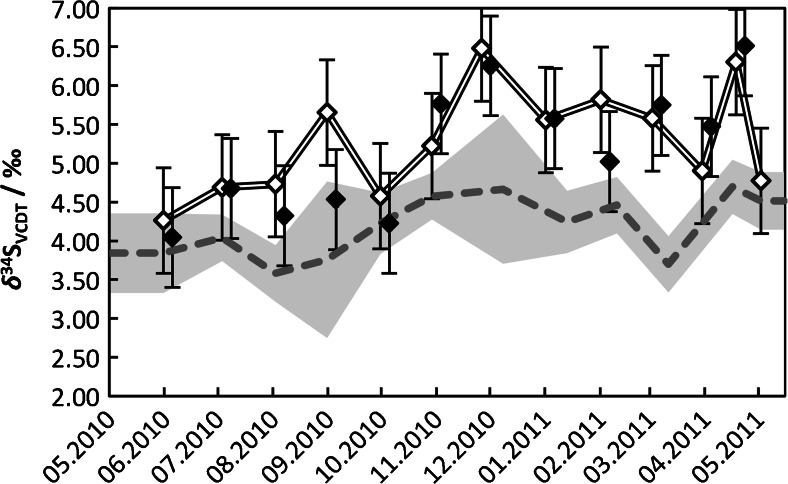
Mean sulfur isotopic composition of rainwater and soil solution sulfate (*n* = 3 for each *data point*). Precipitation (*open diamonds*) corresponds well with throughfall (*black diamonds*). Soil solution (*dashed line*, mean of three soil depths) follows the trend of rainwater. *Error bars* and the *grey area width* represent combined uncertainties

The results are presented as averaged values from the three sampling sites. The *δ*^34^S_VCDT_ of precipitation as well as throughfall samples *δ*^34^S_VCDT_ ranged between 4.0 and 6.5 ‰. The maximum was reached in December 2010 and April 2011. *δ*^34^S_VCDT_ values of soil solution sampled at 10, 30, and 50 cm ranged between 3.6 and 4.7 ‰. No significant difference was determined between different soil depths. Therefore, the *δ*^34^S_VCDT_ values of soil solutions averaged over all three soil depths, precipitation, and throughfall were plotted against sampling months in Fig. 4.

## Discussion

### Matrix effects

MC ICP-MS enables the direct analysis of ^34^S/^32^S ratios in dissolved sulfate. However, sample matrix elements can influence the precision and accuracy of the measurement [7, 14]. This is an important issue especially in soil solutions as they show distinctly higher concentration levels as compared to precipitation samples. Primarily, the high contents of dissolved Ca or K (both reach more than 2.5 mmol L^−1^) might question the applicability of MC ICP-MS for a direct and reliable analysis of ^34^S/^32^S ratio without any matrix effects correction.

Ammonium, Al, Fe, Mg, Mn, Na, organic C, Cl^−^, or PO_4_^3−^ did not cause a significant shift in measured *δ*^34^S_VCDT_ ratios for the concentration ranges found in the investigated samples (see Fig. 1). Contrary to [14], we did not observe a matrix effect caused by Fe in our simulated matrix, although one of our tested Fe/S mass ratios (concentrations of 2 and 159 μmol L^−1^ Fe correspond to Fe/S mass ratios of 0.05 and 4.4, respectively) was above the ratio published by Craddock (Fe/S = 0.9). Addition of Ca, K, and Li resulted in a significant shift in the measured *δ*^34^S_VCDT_ ratios (up to −8 ‰) and led also to a pronounced increase in measurement uncertainty (e.g., a *U* (*k = 2*) of 7.4 ‰ was reached when adding 10 mmol L^−1^ Li). Lin [7] observed a strong effect of Cl^−^ on the *δ*^34^S_VCDT_ measurement when adding NaCl to his in-house S standard. Since our observations were different and NH_4_Cl addition caused no bias in our measurement, we could relate this effect mainly to the presence of Na in the solution, since addition of Na led to decrease of the measured *δ*^34^S_VCDT_ values similarly to [7].

[7] and [13] suggest a matrix-matched bracketing standard to correct for matrix effects. It proved that this is hardly possible in the study of a large number of soil solutions with a high variation of matrix elements. Even though the matrix effect can be approximated by a linear function (starting from Ca/S or K/S mass ratios higher than 5 or Li/S mass ratio of 1), the poor repeatability of the regression curve (39 % on the example of Ca) shows that a simple mathematical correction of matrix effects is not conductive as this correction leads to increased measurement uncertainties. Moreover, the decrease of the signal intensity leads likewise to a significant increase in the measurement uncertainty. The use of an internal standard (e.g., Si) [5, 18] is mainly hampered by the decrease of the analyte signal intensity as well. In addition, it cannot be assumed a priori that internal standard and analyte are subject of the same IIF. As a consequence, a sulfate/matrix separation has proven to be a precondition for accurate S isotope ratio analysis by MC ICP-MS.

### Anion exchange on resin membranes

The applied anion exchange resin on plastic membrane proved its suitability for sulfate sampling from ammonium sulfate solution, as well as from a simulated soil solution matrix. Sulfate was taken up quantitatively by the membrane under the investigated parameter (simulating natural conditions). At the same time, all studied matrix elements (cations and organic carbon) remained completely in the initial solution. Addition of Ca, K, Li, and Na slowed down the exchange rate of sulfate on the resin significantly. As all these elements were added as salt solutions to a S standard, the deceleration can be explained by the presence of dissolved anions (up to 32 mmol L^−1^ NO_3_^−^ when adding 2.5 mmol Ca single-element standard). Due to this observed reduction of anion exchange rates caused by co-dissolved anions and in accordance with literature [9], an exposure time of 16 h was chosen. The anion exchange proved to be robust in a pH range between 2 and 11 which covers well the range which is expected in natural precipitation and soil solution samples.

Kwon et al. tested competitive anion exchange on their plant root simulator [9]. They observed that nitrate occupied a significant portion of the exchange sites and hampered the exchange of sulfate. In our experiments, the sulfate exchange was slowed down significantly first at Cl^−^ and NO_3_^−^ concentration of 5.0 mmol L^−1^, corresponding to Cl^-^/S, resp. NO_3_^−^/S molar ratio of 8.

Due to the large variation of sulfate in natural samples, preconcentration via the anion exchange resin is an asset. Depending on the initial volume and the final elution volume, a significant preconcentration is achievable by the use of an anion exchange membrane under routine laboratory conditions without compromising quantitative S/matrix separation.

Isotope ratio analyses of initial S standard solutions and eluates proved that no significant isotopic fractionation during sulfate separation occurred for both low (40 μmol L^−1^ SO_4_^2−^) and high (1.25 mmol L^−1^ SO_4_^2-^) sulfate concentrations independent of the initial volume/elution volume ratio (see Fig. 3). Separation of sulfate from a simulated matrix was not accompanied by isotopic fractionation either (see Fig. S2). Only NO_3_^−^ and Cl^−^ added together with the investigated matrix elements in concentrations higher than 5 mmol L^−1^ caused a significant fractionation of sulfur stable isotopes during the sulfate separation (see ESM Table S1). This was accompanied with significantly lower recovery (down to 12 % when NO_3_^−^ concentration reached 32 mmol L^−1^). However, such a high anion concentration was not found in any of the more than 1000 analyzed natural water samples (see Table 2). Therefore, we state that the separation technique is suitable for ^34^S/^32^S analysis of dissolved sulfate in natural water samples.

### Precipitation, throughfall, and soil solution samples

The developed method was applied for a study on water samples from forest ecosystems in the Vienna Woods. Wet and dry depositions of atmospheric sulfur are the main sources of sulfate in the environment [10]. Elemental composition of throughfall is given by elements present in precipitation, by material deposited as particles, gases, or cloud droplets being washed off during a precipitation event, and by exchange processes within the canopy (including foliage, woody parts, epiphytes, and microorganisms). Canopy exchange includes both leaching (efflux from the canopy) and uptake or retention (influx to the canopy) [27]. Therefore, precipitation and throughfall were compared in this study. No significant difference in *δ*^34^S_VCDT_ values was observed between the two water types. This indicates that neither dry deposition on the leaf surface nor canopy exchange processes affect the isotopic composition of S significantly. However, S isotope fractionation may be hidden behind the combined uncertainty of the measurement. Higher *δ*^34^S_VCDT_ values during winter months (December) and spring (April) might be caused by the change in emission sources (e.g., elevated central and domestic heating during the winter), because, depending on fuel used, the emitted SO_2_-*δ*^34^S_VCDT_ values can vary strongly [1].

No significant change in soil solution *δ*^34^S_VCDT_ values was observed for different soil depths (10, 30, and 50 cm). From this point of view, the ecosystem seems to be homogeneous within the first 50 cm soil depth in our study sites.

When comparing throughfall with soil solution, lower absolute values of *δ*^34^S_VCDT_ were observed in the soil solutions even though the results overlap within their uncertainties. Depletion in ^34^S of SO_4_^2−^ in soil solution in comparison to SO_4_^2−^ in throughfall may indicate S mineralization as a potential SO_4_^2−^ source, because the soil microflora prefers the lighter ^32^S isotope [11]. Furthermore, it has been suggested for aerobic, forest soils that the mineralization of labile organic S produces SO_4_^2−^ that is more depleted in ^34^S compared to adsorbed SO_4_^2−^ or the SO_4_^2−^ in soil solution. Adsorption/desorption causes no significant isotopic discrimination [1]. The *δ*^34^S_VCDT_ values of this study indicate that the soil solution SO_4_^2−^ budget is driven by throughfall chemistry. A considerable portion of the atmospherically deposited sulfate is cycled through the organic S pool before being released to the soil solution. This cycling is reflected in the abovementioned lower *δ*^34^S_VCDT_ values in the soil solutions. During most of the year, the S isotopic composition of the soil solution follows the pattern of throughfall without substantial delay. Adsorption and desorption are, thus, not important processes within the nutrient-rich (high-pH) soils.

## Electronic supplementary material

ESM 1 (PDF 240 kb)
