# Supplementary material for: MC ICP-MS δ34SVCDT measurement of dissolved sulfate in environmental aqueous samples after matrix separation by means of an anion exchange membrane
Source: Anal Bioanal Chem. 2015 Oct 5;408:399–407. doi: 10.1007/s00216-015-9053-z (PMC4709390; doi:10.1007/s00216-015-9053-z)
Supplement: Supplementary file 1 — (PDF 240 kb) [file 216_2015_9053_MOESM1_ESM.pdf]

Analytical and Bioanalytical Chemistry

Electronic Supplementary Material

MC ICP-MS $\delta^{34}S_{VCDT}$ measurement of dissolved sulfate in environmental aqueous samples after matrix separation by means of an anion exchange membrane

Ondrej Hanousek, Torsten W. Berger, Thomas Prohaska

Table S1

Test type	Concentration / (mmol L^{-1})								Immersion solution		pH	Eluate volume / ml	Matrix element removed / %	Sulfate recovery / %	$\Delta^{34}S_{VCDT}$ / ‰
	SO_4^{2-}	Ca	K	Li	Na	C (organic)	Cl^-	NO_3^-	Volume /ml	Time / h			Average ± *SD*	Average ± *SD*	Average ± *U* (*k*=2)
Sulfate exchange	0.04								30	16	5.6	30		100 ± 0 (*n* = 2)	-
	0.06								15	16	5.6	15		99 ± 3 (*n* = 10)	0.01 ± 0.50
	0.15								30	16	5.6	30		100 ± 1 (*n* = 2)	-0.19 ± 1.47
	1.09								30	16	5.6	30		101 ± 1 (*n* = 2)	-0.44 ± 0.55
Matrix separation	0.16	1.3						16	15	16	5.6	15	100 ± 0	48 ± 4 (*n* = 5)	-1.33 ± 0.82
	0.16	2.5						32	15	16	5.6	15	100 ± 0	12 ± 1 (*n* = 9)	-1.75 ± 1.40
	0.52		4.2				4.2		20	16	5.6	20	100 ± 0	93 ± 2 (*n* = 2)	-0.38 ± 1.11
	0.06			3.5			3.5		15	16	5.6	15	100 ± 0	79 ± 4 (*n* = 3)	-
	0.06			7.3			7.3		15	16	5.6	15	100 ± 0	75 ± 3 (*n* = 3)	-
	0.06				1.9			1.9	15	16	5.6	15	100 ± 0	83 ± 4 (*n* = 3)	-
	0.06				3.8			3.8	15	16	5.6	15	100 ± 0	79 ± 2 (*n* = 3)	-
	0.62					11.6			20	16	5.6	20	97 ± 0	91 ± 1 (*n* = 2)	-
Anion competition	0.57						0.1	0.1	15	16	5.6	15		96 ± 4 (*n* = 3)	-
	0.57						1.0	1.0	15	16	5.6	15		101 ± 2 (*n* = 3)	-
	0.57						5.0	5.0	15	16	5.6	15		99 ± 2 (*n* = 3)	-
	0.57						10	10	15	16	5.6	15		67 ± 2 (*n* = 3)	-
	0.57						15	15	15	16	5.6	15		56 ± 5 (*n* = 3)	-
pH	0.06								15	16	1.1	15		20 ± 1 (*n* = 2)	-
	0.06								15	16	1.4	15		51 ± 0 (*n* = 2)	-
	0.06								15	16	2.0	15		93 ± 1 (*n* = 2)	-
	0.06								15	16	11.4	15		93 ± 1 (*n* = 2)	-
	0.06								15	16	11.9	15		91 ± 1 (*n* = 2)	-
	0.06								15	16	12.6	15		78 ± 4 (*n* = 2)	-
Kinetics	0.88								15	1/6	5.6	15		74 ± 2 (*n* = 2)	-
	0.88								15	1/2	5.6	15		95 ± 4 (*n* = 2)	-
	0.88								15	1	5.6	15		100 ± 0 (*n* = 2)	-
	0.88								15	2	5.6	15		100 ± 0 (*n* = 2)	-
	0.88								15	4	5.6	15		100 ± 0 (*n* = 2)	-
	0.88								15	16	5.6	15		100 ± 0 (*n* = 2)	-
Preconcentration	0.06								15	16	5.6	10		149 ± 3 (*n* = 2)	-0.09 ± 0.60
	0.06								15	16	5.6	5		284 ± 2 (*n* = 3)	0.04 ± 0.71

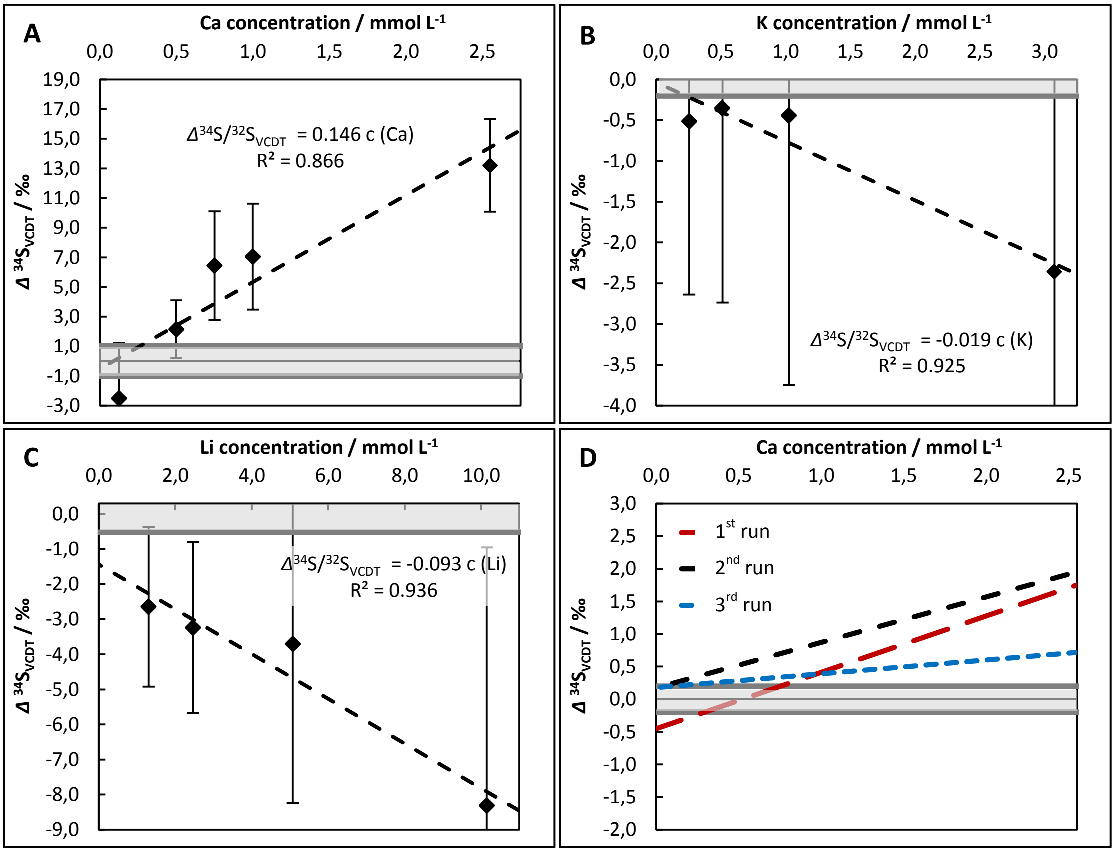

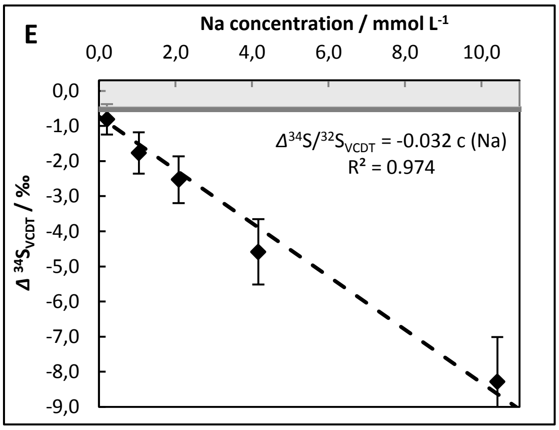

Fig. S1 The influence of addition of matrix elements (A: Ca, B: K, C: Li and E: Na) at increasing concentration to a S standard on measured $\delta^{34}S_{VCDT}$ values. The $\Delta^{34}S_{VCDT}$ represents a relative shift from the corresponding reference value (grey range). Error bars represent the expanded uncertainty *U* (*k* = 2). The figure D shows the repeatability of the relationship between increasing Ca concentration and measured $\delta^{34}S_{VCDT}$ values (all three runs were performed within one measurement session under the same measurement conditions).

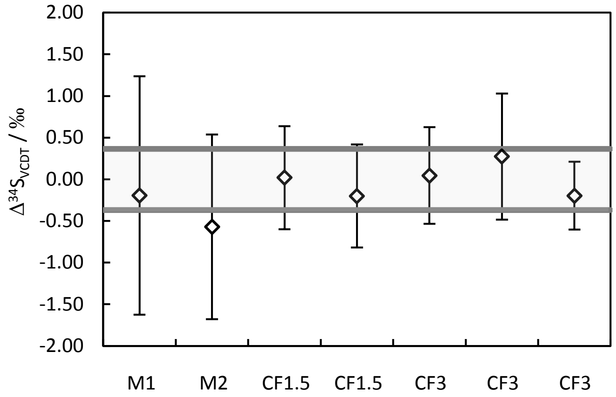

Fig. S2 $\delta^{34}S_{VCDT}$ measured after separation of sulfate from a simulated matrix solution by the anion exchange membrane (M1, M2) and after sulfate enrichment by the membrane (CF1.5 and CF3 for concentration factors 1.5 and 3, respectively). The data is presented as a relative shift ($\Delta^{34}S_{VCDT}$) from the initial solution (grey range). Error bars represent expanded uncertainties U (k=2).
